# WHO informal consultation on regulatory considerations for evaluation of the quality, safety and efficacy of RNA-based prophylactic vaccines for infectious diseases, 20–22 April 2021

**DOI:** 10.1080/22221751.2022.2026742

**Published:** 2022-01-31

**Authors:** Margaret A. Liu, Tiequn Zhou, Rebecca L. Sheets, Heidi Meyer, Ivana Knezevic

**Affiliations:** a ProTherImmune, Lafayette, CA, USA; b Department of Health Product Policy and Standards, World Health Organization, Geneva, Switzerland; c Independent Expert, Silver Spring, MD, USA; d Paul-Ehrlich-Institut, Langen, Germany

**Keywords:** WHO, mRNA vaccines, prophylactic vaccines, infectious diseases, regulatory considerations, COVID-19

## Abstract

This paper presents the key outcomes of the above WHO informal consultation with global stakeholders including regulatory authorities, vaccine developers and manufacturers, academia and other international health organizations and institutions involved in the development, evaluation and use of messenger RNA (mRNA) vaccines. The aim of the consultation was to further clarify the main principles to be presented in an upcoming WHO guidance document on the regulatory considerations in evaluating the quality, safety and efficacy of mRNA prophylactic vaccines for infectious diseases. This WHO guidance document is intended to facilitate global mRNA vaccine development and regulatory convergence in the assessment of such vaccines. The urgent need to develop such a document as a new WHO written standard is outlined in this report along with the key scientific and regulatory challenges. A number of key conclusions are provided at the end of this report along with an update on the steps taken following this meeting.

## 1. Introduction

The World Health Organization (WHO) provides technical guidance as part of promoting regulatory convergence among its Member States in order to assure the quality, safety and efficacy of biological products, including vaccines. This includes the development of safe and efficacious vaccines for use against emerging infectious diseases in the context of preparing for and responding to public health emergencies (PHEs).

mRNA vaccines have been under development for nearly 30 years but due to challenges related to production, stability and reactogenicity, it has mainly been in the past two decades that advances have been made enabling them to enter into clinical trials. Importantly, the COVID-19 pandemic has necessitated a significant acceleration of development efforts, resulting in an impressive proof of concept for their efficacy and safety as prophylactic vaccines for COVID-19. The demonstrated utility of mRNA vaccines to respond to a PHE, and the need for global usage and large-scale manufacturing, have validated the WHO’s decision to develop guidance for this class of vaccines and have resulted in a significant acceleration of the work.

The WHO Expert Committee on Biological Standardization (ECBS) discussed these issues at its meetings in August and December 2020 [1, 2] and supported the development of a WHO document on regulatory considerations in the evaluation of the quality, safety and efficacy of mRNA-based prophylactic vaccines for infectious diseases (hereafter “WHO document”), which could be updated as more scientific and clinical data became available. WHO initiated the work in 2020 and set up a drafting group composed of individuals with expertise in mRNA vaccines and their regulation to prepare a series of draft versions of the WHO document. A first draft was posted on the WHO website for first round public consultation from 22 December 2020 to 31 January 2021 and the public was invited to comment during this time. The WHO then organized an informal consultation, which was held virtually on 20–22 April 2021. In attendance were around 90 participants from 24 countries across the six WHO regions. These included experts and representatives of stakeholders from industry, academia and other research and clinical entities, regulators and other international health agencies. Regulators from 20 countries took part in the consultation and provided useful input regarding the regulatory aspects of the vaccine evaluation.

Dr Clive Ondari (WHO headquarters (HQ), Switzerland) welcomed the meeting participants and highlighted both the potential of mRNA vaccines and the need for international regulatory convergence in their development and use. He emphasized the need to ensure coordination of their development and regulatory considerations, underscoring that these would need to be updated as more information became available.

Dr Heidi Meyer (Paul-Ehrlich-Institut, Germany) chaired the consultation; Dr Margaret Liu (WHO consultant, USA) served as rapporteur and Dr Rebecca Sheets (WHO consultant, USA) was the moderator for the discussions on the revisions to the draft document.

Dr Tiequn Zhou (WHO HQ, Switzerland) provided the background, objectives and anticipated outcomes of the consultation. As a potential platform technology, nucleic-acid-based vaccines may enable a rapid response to the priority diseases listed in the WHO R&D Blueprint of prioritized diseases in public health emergency contexts [3]. She underscored the rapid pace of mRNA vaccine development, with those for prophylaxis against COVID-19 having been authorized for emergency use and/or given conditional marketing authorization by National Regulatory Authorities (NRAs) and by WHO Emergency Use Listing (EUL) since December 2020. However, gaps still exist in the understanding of the mechanisms of action and long-term performance of such vaccines, including in terms of their safety and efficacy. Dr Zhou underscored that this is a fast-evolving field with both ongoing studies and the need for future studies.

Dr Zhou described the process and progress to date in developing the WHO guidance document on the evaluation of mRNA vaccines. During the public consultation on the first draft document, a considerable number of comments were received, reflecting the level of high interest among the public. The drafting group reviewed and discussed the comments, prepared the second draft and identified key issues for broader discussion. The objectives of the current meeting were to: (a) review the global pipeline of prophylactic mRNA vaccine development including for COVID-19; (b) exchange experiences and perspectives among researchers, manufacturers and regulators regarding aspects relevant to the quality, safety and efficacy of the mRNA vaccines; and (c) review the second draft of the WHO document, discussing key issues identified during the public consultation and issues on standardization. It was expected that a consensus would be reached, and improvements would be proposed on the further development of the document prior to its submission to the ECBS.

Dr Ivana Knezevic (WHO HQ, Switzerland) then gave an update on the biological standardization activities of the WHO. She described the WHO written and measurement standards for vaccines, biological therapeutic products, and cell and gene-therapy products that are publicly available [4]. She explained that WHO written standards are intended to: (a) provide key principles for the evaluation of biologicals as a basis for setting national requirements and for WHO prequalification; (b) leave space for NRAs to formulate additional/more-specific requirements; (c) act as living documents that will be developed further in line with progress in scientific knowledge and experience; (d) assist with the implementation of the guidelines into regulatory and manufacturing practices through global, regional and national workshops involving regulators, manufacturers and other relevant experts, as well as training and advisory groups; and (e) consider guidance issued by other bodies – the intention being to complement them, not to create conflicting guidance. When describing the global measurement standards, which are key elements for product development and licensing, Dr Knezevic highlighted three WHO International reference preparations for SARS-CoV-2 adopted by the ECBS in December 2020 [2] namely: the First WHO International Standard for SARS-CoV-2 RNA for NAT-based assays, the First WHO international standard for anti-SARS-CoV-2 immunoglobulin, and the First WHO International Reference Panel for anti-SARS-CoV-2 immunoglobulin panel. These standards aim to facilitate the development, validation and assessment of molecular and antibody assays, facilitate the comparability of results from different assays/laboratories and help harmonize the evaluation of diagnostics, vaccines and other products.

Dr Knezevic underscored the focus to be placed on scientific evidence followed by the WHO consultation process in further developing the guiding principles for evaluating the quality, safety and efficacy of mRNA vaccines [5].

## 2. Updates on global development of prophylactic mRNA vaccines for infectious diseases

Dr Margaret A. Liu provided an overview of mRNA vaccine technologies and the global pipeline to remind participants of the developments for both the nucleic acid components (i.e. modifications of the mRNA itself) as well as the lipid nanoparticle (LNP) formulations which have been made over the past several decades. Dr Liu described the advances made to increase the stability of mRNA vaccines, to increase the amount of antigen translated from the mRNA and the duration of antigen expression, and modifications that have optimized the immune responses, including decreasing the undesired types of immune stimulation. Explanations of the differences between traditional and self-amplifying mRNA were provided along with definitions of terminology. While other formulations are under development, only LNPs are addressed in the WHO document since they are the ones currently used in clinical entities. Knowledge gaps and challenges were discussed including: the applicability/efficacy for other diseases with different pathophysiology (e.g. HIV, tuberculosis), the duration of protection, the utility for diseases where a single-dose vaccine is desired (e.g. Ebola), boostability in the face of strain mutations, the tolerability/acceptability of adverse effects for non-pandemic diseases, and continued demonstration of safety with increased utilization (e.g. anaphylaxis, possible differences of adverse events for different populations). Note was made of the potential limitations for global use based on the cost of manufacturing and concerns of limitations for use in resource-constrained settings with current thermostability limitations.

Dr Nick Jackson (Coalition for Epidemic Preparedness Innovations (CEPI), UK) provided the context for mRNA vaccine development during the COVID-19 pandemic, describing progress of SARS-CoV-2 mRNA/LNP vaccines, highlighted challenges ahead for mRNA, describing lessons learned and how that has informed what will be done for future pandemic situations. He noted that COVID-19 has driven the application of over three dozen mRNA platforms as candidate vaccines. Dr Jackson listed challenges that lie ahead for mRNA-LNP technologies including productivity, thermal stability, manufacturing footprint, and improvement of tolerability. The price per dose of mRNA vaccines is greater than for other vaccines such as the COVID-19 adeno-vectored vaccines [6]. Very specific aspects of the manufacturing drive the productivity and hence the cost, including the amount of RNA per dose, the process scale, production yield, the cost of the 5’ cap analogues, downstream purification losses, raw material recycling and capital investment costs. Scale-up and scale-out of RNA production has also exposed limitations in the supply of suitable quality raw materials, which include DNA templates, enzymes, nucleotides, capping agents and LNP components. While RNA is scalable as a platform, the footprint for mRNA manufacturing capabilities remains limited globally. Thermal stability remains a challenge. Dr Jackson presented examples of potential future indications for mRNA/LNP vaccines that may include adapting vaccines to new strains or making vaccines with broad protection against diverse strains, targeting proteins which are difficult to manufacture by recombinant technologies. Other potential areas of development include vaccines against diseases for which a higher financial investment is needed and/or diseases for which a rapid response is needed. In the latter case, it might be necessary to establish prototypical libraries of mRNA encoding vaccine antigens and to explore the efficacy of single-dose vaccines.

## 3. Experiences and perspectives from developers and manufacturers

Dr Ruben Rizzi and Dr Andreas Kuhn (BioNTech, Germany) presented their experience in development, manufacturing, quality control, and nonclinical and clinical aspects for the BioNTech /Pfizer mRNA COVID-19 vaccine. For the mRNA types tested in Phase 1 and 2 clinical studies, the manufacturing processes used for RNA and LNP production are essentially identical for all candidates, and are generally applicable to a wide range of RNAs with respect to sequence and size; i.e. a platform approach is used. The company-wide leveraged platforms, early engagement with the regulatory authorities and regulatory flexibilities are key to the rapid clinical development of mRNA COVID-19 vaccines.

Dr Florian Neske (CureVac, Germany) described their COVID-19 vaccines using natural nucleotides with sequence optimization. He presented a schematic of the manufacturing process including starting materials, in-process controls (including intermediates) and release testing of final drug products. Biodistribution may change with different formulations. Key quality control considerations were described. Dr Neske raised the point that because of the limited number of available mRNA manufacturing descriptions at the time of writing the draft WHO document, some of the requirements may not be needed for alternative manufacturing processes. He suggested the WHO document should include case-by-case statement to avoid restricting future vaccine development and regulatory considerations.

Dr Don Parsons (Moderna, USA) described the general process for making their mRNA vaccines, including the LNP, as a co-component of the active substance. He proposed that a flexible approach should be taken to the definition of final formulated bulk, accommodating that the bulk substance may be formulated (e.g. encapsulated in LNP) but then stored concentrated in comparison to what is later diluted and filled for the final formulated vaccine. Dr Jacqueline Miller (Moderna, USA) described the immunological mechanisms of mRNA vaccines and gave a list of the Moderna prophylactic vaccine candidates. The same LNP was utilized across the platform, so she stated that, for a mature platform, a platform approach may allow aspects of the quality, nonclinical and clinical development to be standardized, such as evaluation of genotoxicity and biodistribution.

Dr Bo Ying (Abogen, China) introduced their mRNA platform for targeting cancer, protein replacement therapy, and vaccines for infectious diseases. He gave an overview of the mRNA and LNP production processes and in-process controls. He pointed out that capping efficiency will affect the safety and efficacy of mRNA vaccines and that purity, tail length and distribution will affect the efficiency of translating mRNA into proteins. He raised issues about acceptance of minor changes for platform technology, regulation of mRNA vaccines against variants, and multivalent vaccines.

## 4. Regulatory perspectives

Dr Keith Peden (Center for Biologics Evaluation and Research (CBER), Food and Drug Administration (FDA), USA) presented the FDA’s experience with mRNA vaccines, including product and Chemistry, Manufacturing and Controls (CMC) issues, potency determination, pre-clinical studies, efficacy assessment (what to monitor and what assays to use), evaluation of possible vaccine-enhanced disease, and the question of whether or not mRNA can be viewed as a platform technology. He commented that whether the individual LNP component should be evaluated separately or as the vaccine is an individual NRA’s decision. CBER decided only the product should be tested. The issue of whether mRNA vaccines are a platform technology and what the implications would be if so, has been discussed at the FDA. This has implications, e.g., what testing would be required for a new mRNA that expresses a new antigen using the same LNP and manufacturing process? What pre-clinical studies would be required, and which could be dispensed with based on data from similar products? Could the vaccine development process be streamlined? CBER has determined that this is in flux, and has not required that biodistribution studies be performed on a new vaccine if studies with another vaccine using the same manufacturing process and same LNP have already been done. It is expected that modifications to the manufacturing process, and likely the encapsulating lipids will occur in the future.

Dr Jiaqi Lu (Centre for Drug Evaluation, National Medical Products Administration, China) provided an overview of China’s regulatory guidelines on COVID-19 vaccines and on the CMC of mRNA vaccines and then presented a CMC evaluation strategy of mRNA vaccines by NMPA. A key point was that the national guideline specified that it only reflected the current knowledge and opinions of mRNA vaccines noting that it will be updated as research progresses and scientific knowledge increases. Dr Lu discussed challenges and perspectives including comparability studies in case of manufacturing changes such as scale-up/scale-out, changes of manufacturing site and equipment, and changes of suppliers of excipients. Specifications for purity, particle size, encapsulation efficiency and potency (in vitro, in vivo) are additional issues. Another challenge is whether this can be a platform technology to quickly respond to virus variants, possibly optimizing antigen sequences or making multivalent vaccines, and to be able to accelerate the development of mRNA vaccines based on experience with the manufacturing process, formulation, characterization and stability, with an acknowledgement of relative risks.

Dr Ka-Wai Wan (Medicines and Healthcare products Regulatory Agency, UK) provided an overview including general considerations and specific issues concerning quality, nonclinical and clinical assessment of mRNA vaccines to assure their quality, safety and efficacy. For COVID-19, additional challenges included the novelty of the coronavirus and being the first mRNA vaccines to be authorized globally, and an accelerated process due to the PHE. She acknowledged that some decisions made were on the basis of risk in the context of a PHE and would likely not be the same for other new vaccines in development. Dr Wan detailed a number of issues regarding safety for which there are gaps in knowledge, asking whether these gaps are acceptable. Examples include information about how novel components are cleared from the body, and over what time course, whether novel components cross the placenta, a lack of correlate of protection for COVID-19, and the durability of the immune response.

## 5. Discussion on the draft WHO document

Days two and three of the meeting were devoted to reviewing the draft WHO document. The discussion was moderated by Dr Rebecca Sheets, who started by providing an outline of the current draft, a summary of comments received from the first round of public consultation, and the main issues to be discussed at this meeting. The group agreed upon a framework for the approach to developing the final document as shown in Box 1.

Box 1

**Table:** 

**Consensus of overarching considerations that will inform the final document** Because new data are rapidly accruing, this WHO document may need to be updated in the future when more information is available. To avoid restricting future development, this WHO document should include statements on the need for case-by-case considerations and benefit/risk evaluation. It is important that NRAs are engaged early on in vaccine development and evaluation to ensure the optimal progress of clinical trials of mRNA vaccines.

Significant time was spent discussing major issues, shown in Box 2, as these were foundational concepts for the document.

Box 2

**Table:** 

**Major Issues raised in the discussion and consensus reached:** Key discussions centred on the concept of mRNA as a platform technology, what should be accepted for various assays/characterizations including: potency, safety, toxicity studies, biodistribution, mechanism of action studies, variants/multivalent vaccines, etc. These aspects were each specifically discussed during the review of the relevant section(s) of the document. Specific manufacturing issues that guide the writing and revisions: Leveraging prior/existing experience with the same platform technology will facilitate expeditious development of mRNA vaccines in response to future emerging pathogens. Technologies/processes for mRNA vaccine production are not all the same among manufacturers, which has implications on the differing approaches to quality control and evaluation. Detailed information about mRNA vaccine production and quality control is either not yet available, or is proprietary. There is currently limited experience and lack of a “gold/or harmonized standard.” Flexible approaches are suggested by manufacturers given the ongoing development of the technology and lack of existing standards.

For the actual wording of the document, consensus was reached or revisions and additional text were proposed, when necessary. A summary of the decisions regarding the various issues was presented by the rapporteur (Dr Margaret Liu) at the end of the meeting to ensure that the participants agreed with the decisions and proposed changes or areas for further effort by the Drafting Group. The proposed amendments, revisions or other changes to the draft WHO document are summarized in Table 1.

**Table 1.** Summary of major proposed changes for the draft WHO document by sections.

Section	Proposed changes
Introduction Background Purpose and scope Background Purpose and scope Terminology General considerations Manufacture and control of mRNA vaccines Nonclinical evaluation Clinical evaluation	§ Various points of clarification, specific wording and changes in organization were agreed upon. Such changes occurred throughout the document, but particularly in these sections, which provided the foundational information for the document. § Add following additional information as deemed important for the nonclinical and clinical evaluation sections: expression efficiency of self-amplifying mRNA compared with standard mRNA constructs further information about LNP relevant to their in vivo behaviour including the cells to which they target and effects on innate immunity A request from a reviewer during the first public consultation to list the nucleotides generally used for mRNA vaccines was rejected as not only would the list be too long, but also new ones might be used in the future. § Because some pathogens may have different strains, or variants may arise, the issues related to strain changes and/or increased valency may need to be considered in vaccine composition. Guidance and reference to WHO documents dealing with variants and multivalent vaccines are provided throughout the document. § Consensus was reached for the following terms: “Drug substance” refers to mRNA “Final bulk” was changed to “final formulated bulk” and the definition has been amended to state that the final bulk may be stored at a higher concentration and diluted prior to fill “Final vaccine” aka “drug product” was clarified to include “mRNA formulated in LNP” for this document “Platform technology” was a newly added term and prompted extensive discussion due to considerations such as whether a licensed product must form the basis for a platform technology as well as what defines the technology (i.e. the LNP-formulated mRNA, and any impact of changes to the mRNA or the LNP, etc.). Continuing discussions by the drafting group will propose a definition in the next draft. Further issues related to considerations of a platform technology are discussed in the subsequent sections in relationship to general considerations for multivalent/multi-strain vaccines and for nonclinical and clinical evaluation. Moreover, the issues addressed regarding the implications of a platform technology concept required additional discussions and amendments under other sections such as “Manufacture and control of mRNA vaccines” and the nonclinical and clinical evaluation sections. § Multivalent candidates may be generated for vaccines targeting different strains of the same pathogen or for vaccines targeting more than one pathogen. The drafting group is working on modifications to the language regarding this point, including referencing existing WHO guidelines on clinical evaluation of vaccines [7] as discussed more extensively in that section. § Specific manufacturing issues that will guide the writing and revisions include: Leveraging existing experience with the same platform technology will facilitate expeditious development of mRNA vaccines in response to future emerging pathogens. The need to clarify the definition of “platform technology” was raised for this section as well. A key point was made that the technologies and components differ between manufacturers. For example, in addition to the differences between standard and self-amplifying mRNAs, certain manufacturers use modified nucleosides, whereas others modify the sequence of the mRNA from that of the natural pathogen antigen but use native nucleosides. Each manufacturer also has developed their own LNP based on differing lipids, with differing modifications, and their own process for formulation of the mRNA into the LNP. This has implications on the quality control and evaluation. Currently, there is limited experience and a lack of a “gold/or harmonized standard.” Based on the different components and methodologies, it might not be possible to have such a standard, even for vaccines against the same disease. Certain information about production and quality control methods and specifications is also confidential since it is based on a given manufacturer’s proprietary platform technology. Flexible approaches about what should be included for specific quality-control testing were suggested by manufacturers to take into account the ongoing development of the technology, e.g. confirmation or measurement of poly(A) tail length might not be needed on a lot-by-lot basis if encoded into the DNA template instead of added enzymatically. § While assessments for typical parameters such as content, identity, purity, mRNA integrity, potency, other quality and safety parameters, and stability would be needed, it was not recommended to provide a specific list of required assays, since the current situation is not standardized and the technologies may evolve. Certain tests might be for characterizations vs. control or release tests. A table of examples of assays suitable for various purposes was added to the document. § The use of starting materials that are appropriate for the stage of development of the product might mean that under emergency conditions, one might accept divergence from the otherwise expected full compliance with good manufacturing practices (GMP). This is an example of balancing a trade-off of risk and benefit in an emergency setting, such as with COVID-19. § For self-amplifying mRNAs where the replicon is encoded by a separate mRNA, additional controls may be needed to ensure adequate encapsulation of the (two) mRNAs, potentially different expression of the encoded proteins, and the ensuing impact on the potentially different safety and efficacy of the vaccines based on self-amplifying mRNA versus mRNA. § The table of analytical methods was re-labelled to clarify that the listed assays were examples rather than specifically recommended assays; the statement in the narrative leading into the table was also modified. § Differences of opinion arose as to what should be required for certain control parameters. One example is what would be an indication of vaccine potency, and in particular whether an in vivo assay would be needed or if an in vitro assay would be adequate or preferred. Language was changed in the potency section to soften the statement about application of the 3Rs (Replacement, Reduction and Refinement) approach, which had stated that it was recommended to avoid animal-based potency methods. § Different types of impurities may be seen and different products may have different properties. Specifications, including upper limits, need to be set on a case-by-case basis, for example depending on the length of the mRNA. A suggestion was made to include a statement that limits should be reflected by clinical batch data. § Regarding the issues of whether modified or nonmodified nucleosides are used together or with complete replacement, the wording was changed to reflect different mRNA designs. Wording suggested may include, “in cases where specific ratios or positions are part of the product design.” The text was clarified that at present, when modified nucleosides are used, they entirely replace the natural nucleoside. § A comment was also added about double-stranded RNA: “Testing for dsRNA needs to be done depending on the process and its ability to generate it.” dsRNA can be generated during certain in vitro transcription manufacturing processes of mRNA. This impurity can stimulate innate immune responses, and thus should be removed or quantitated and controlled for if the manufacturing method produces it. § The text was amended to specifically mention that information about all the components of the vaccine, i.e. LNP and excipients as well as the mRNA would need to be provided. This includes the rationale for their inclusion as well as their specifications. § In addition, the document will be amended to additionally address LNP controls regarding manufacture, purity, consistency and purification to remove excess raw materials. Section on “Manufacture and control of lipid nanoparticles and encapsulation of mRNA” needs more attention to add controls. Examples of issues that need to be addressed include the fact that the size of the mRNA can affect its interaction with the LNP and the concepts of same/identical vs. comparable/essentially unchanged in regard to what could be considered a platform technology. § A requirement was added that information related to the generation of the linearized DNA template will also be necessary, including the cell banks, stability and other characteristics. § Additional discussions dealt with identity, purity, quantification and physical state, and additional quality parameters (e.g. poly(A) tail length, degree of capping efficacy) resulting in ongoing modifications to the text. § The section on reference standards was discussed and expanded to address additional issues such as the role of the NRA, a standards programme, and the conditions for formulation and storage. § Additional attention was deemed necessary for issues related to the impact of changed mRNA upon the LNP. § The labelling recommendations were discussed and expanded. § Safety and toxicity: Discussions centred around biodistribution, persistence and inflammation of both the mRNA and the LNPs. § Because novel lipids and novel formulations can affect the charge of the LNP, a discussion ensued regarding which component needed genotoxicity and systemic toxicity studies. Much as would be done for a novel adjuvant, the novel lipids or formulation might be included as a study arm in comparison with the vaccine, in such studies. Reference to relevant WHO and International Council for Harmonization guidelines [8,9] is made. § As a result of the discussion on the rationale that integration studies are not necessary for mRNA vaccines, the text was amended to read: “Further, the design of candidate mRNA vaccines should be considered so that they do not include specific RNA-binding sites for primers required for the reverse transcriptase to initiate transcription,” to specifically highlight that the vaccine should be designed to exclude such RNA-binding sites. § The possibility of accelerating the nonclinical evaluation of mRNA vaccines in the case of strain changes when all other aspects of the construct, manufacturing processes and controls are the same was discussed with modifications to the text proposed. § The types and scope of adverse effects including immunological parameters, and how these could affect the design of clinical trials was discussed with reference to existing WHO guidance documents. § Efficacy evaluation in a public health emergency when variants arise, and the possibility of bridging studies, were further discussed and modifications to the section are under consideration with reference made to existing WHO guidelines. A caution was made by some participants that this document should provide general guidance and specific considerations will be case-by-case. Particularly, there are other groups working on regulatory guidance on strain changes and immunobridging for COVID-19 vaccines, and there is established practice for global annual influenza vaccine strain changes, which should not be contradicted by this document.

## 6. Conclusions and post-meeting update

The development of the WHO regulatory considerations document was strongly welcomed by stakeholders. Following the informal consultation, the draft document, which had already been modified extensively in real time during the discussions, was further edited to add in the requested additional information and to improve the language to better capture the intention of the suggestions. It was agreed that where consensus had not been reached (for example, on potency assays), discussions would continue and further comments would be expected during the upcoming round of public consultation.

The subsequent draft document was then posted on WHO website in early July for a second round of public consultation until mid-September 2021. Given the considerable level of interest in developing mRNA vaccines against COVID-19, the two rounds of public consultation resulted in numerous comments. Most of these comments were accepted but some were rejected because they were too COVID-19 specific or were already covered by other WHO guidance documents. The drafting group analyzed all comments received and proposed further changes. The resulting document, along with the key issues arising from the public consultations, were reviewed by the ECBS at its meeting of 18–22 October 2021. Specific issues addressed included: (1) refining the definition of a “platform technology,” (2) use of the term “drug substance” and “drug product” instead of “antigen” and “final vaccine” respectively, (3) definition of linear DNA as the starting material, (4) application of GMP for biologicals, (5) potency testing, (6) vaccine labelling and (7) dosing of mRNA vaccines. Having addressed the specific comments raised by the drafting group, the ECBS reviewed the entire document and made further suggestions. Among these, the Committee suggested two further definitions be added to the terminology section: “design of experiments” and “engineering run.” The Committee then adopted the document with the suggested amendments [10]. The resultant document was published on the WHO website [11] to ensure its prompt availability prior to its formal publication in the WHO Technical Report Series.
